# Supplementary material for: Temperate infection in a virus–host system previously known for virulent dynamics
Source: Nat Commun. 2020 Sep 15;11:4626. doi: 10.1038/s41467-020-18078-4 (PMC7493887; doi:10.1038/s41467-020-18078-4)
Supplement: Supplementary file 1 — Supplementary Information [file 41467_2020_18078_MOESM1_ESM.pdf]

Supplementary Information for Knowles et al. *'Temperate infection in a virus-host system previously known for virulent dynamics'*.

Supplementary Note 1: Model equations and description

To understand better the mechanisms underlying the host-viral dynamics we observed experimentally, we studied three versions of a host-virus interaction model:

i) Virulent model: A classic version in which the virus is purely virulent.

ii) Phenomenological temperate model: A modified version of the classic model in which a switch from temperate to virulent mode occurs at specific times, which are informed by the host physiological data obtained in our experiments.

iii) Self-regulated temperate model: A modified version of the phenomenological temperate model that includes suggested mechanisms for the self-regulation of the switch from temperate to virulent infection.

The comparison between model i and ii aims at understanding whether lysis suffices to explain the observed behavior, or temperateness is required to that end. The comparison between models ii and iii aims at understanding whether the mechanisms suggested in the latter provide induction times compatible with those we observed experimentally, and therefore curves resembling those obtained with ii. We explain below all the different assumptions and model terms behind each model version. For all versions, we kept track of the dynamics of healthy hosts, infected hosts, and free/infective viruses.

To present the model equations in a compact way, let us write all the different versions of the model together and differentiate the elements contributed by each version with specific colors. The equations that represent the growth of the uninfected population are presented in black, the additions required to complete a model in which the virus is purely virulent are presented in red, and the additions required to represent temperate dynamics are presented in blue. In other words, the virulent, purely-lytic version of the model uses black and red terms, whereas the temperate versions use all three colors

(additional conditions are imposed for the self-regulated model, see section *Self-regulated temperate version: dynamic switch*).

a) *Uninfected population growth rate*:

All versions of the model use a phenomenological implementation of the growth rate. Using as a reference the experimental data for the uninfected case, in which the host follows an approximately logistic growth, we devised an expression for the growth rate that would produce a logistic curve resembling our population density data in the absence of viruses. The following expression provides a good approximation to how the uninfected population growth rate changed with time (see **Supplementary Figure 3a**):

$$\mu(t) = \mu_{eff}(t)\left(1 - \frac{[H](t) + (1 - r_s)[I](t)}{K}\right) \quad (1)$$

where [*H*] represents the density of uninfected hosts, [*I*] the density of infected hosts (which contribute to resource uptake only before induction, see below), *K* the carrying capacity (see **Supplementary Table 2** for parameter values and units), and:

$$\mu_{eff}(t) = \begin{cases} \mu_{max} & \text{if} \quad t < 2 \\ s_\mu t + n_\mu & \text{if} \quad 2 < t < t_\mu \\ \mu_{min} & \text{if} \quad t > t_\mu \end{cases} \quad (2)$$

that is, the growth rate stays at a maximum level for two days, then decreases linearly to reach a minimum level at t_μ. The parameters of the (decreasing) linear relationship are thus given by:

$$s_\mu = \frac{\mu_{min} - \mu_{max}}{t_\mu - 2}$$

$$n_\mu = \mu_{max} - 2s_\mu = \frac{t_\mu \mu_{max} - 2\mu_{min}}{t_\mu - 2} \quad (3)$$

Eqs.(1)-(3) aim to replicate as closely as possible the growth conditions for the uninfected host population, including unknown/uncharacterized sources of physiological stress for which we may have no information. However, our results do not change qualitatively if we replace Equation (1) for a standard Monod growth function [1] dependent on, *e.g.*, nitrogen as single source of growth limitation in our focal batch experiment.

b) *Model equations*

If [*H*] represents the concentration of uninfected hosts, [*V*] the concentration of extracellular (infective) viruses, and [*I*] the concentration of infected hosts, all in units of individuals per liter (see **Supplementary Table 2**), the dynamics of the system are described by:

$$\frac{d[H](t)}{dt} = \mu(t)[H] - m[H] - k[H][V] + \mu_I(t)[I] \quad (4)$$

$$\frac{d[V](t)}{dt} = r_s B k_L[I] - k([H]+[I])[V] - m_V[V] \quad (5)$$

$$\frac{d[I](t)}{dt} = k[H][V] - r_s k_L[I] - m[I] \quad (6)$$

In the first equation (dynamics of the uninfected host population), the first term represents population growth; the second term represents natural mortality; the third term represents infection events, which occur at a rate *k* (viral adsorption rate); and the last term the fact that infected hosts can reproduce if the infecting virus is temperate which, for simplicity, we assume results in new uninfected hosts. In the second equation (dynamics of the freely-diffusing viral population), the third term represents viral decay out of the host; the second term represents infection events (including the possibility of an infected host to be re-infected by other viral individuals *via* superinfection); and the first term represents the viral offspring resulting from lysis (which only occurs if the virus is purely virulent or has switched from temperate to virulent, see below); each virus

produces *B* virions per host, and we assume here that the offspring is released at a lytic rate k_L (inverse of the latent period, *L*). The latter assumption, which implies that the offspring is continuously released to complete burst size release in *L* days, aims at capturing intraspecific variability in the exact timing of offspring release as well as the possibility of virion release via budding (a possibility for EhV [2]). In the last equation (dynamics of infected hosts), the first term represents infection events; the second term represents lysis of hosts (by either purely viruses or temperate viruses that have switched to virulent mode); and the third term represents host natural mortality.

c) Induction of temperate viruses (i.e., switch from temperate to virulent mode):

The potential temperate mode for the virus is implemented in the equations above *via* a switch function:

$$r_s = \begin{cases} 1 & \text{if virusis virulent} \\ 0 & \text{if virus is temperate} \end{cases} \qquad (7)$$

Following the experimental data, in the temperate versions of the model we assumed that the default mode of the virus is temperate, with a physiologically-dependent switch to virulent mode that we modeled in two different ways.

Simple temperate version: data-informed switch

In this version of the model, a data-informed switch determines the change from temperate to virulent. Specifically, we assumed that such a mode change was triggered by the physiological stress of host cells measured in our experiments. Understanding as stress the decline that healthy hosts show in the photosynthetic performance curve (**Figure 2c**), we imposed in this simple temperate version that induction occur at a particular time, t_s, matching the beginning of the decline in the F_v/F_m curve.

Thus, in this version of the model, $r_s = 1$ for $t \geq t_s$, and zero otherwise. This implicitly assumes that the virus does not switch back to the temperate mode in the duration of the experiment, which is consistent with our experimental results for the

phenomenology of interest, namely the initial increase and decline of the host population. The specific times, t_s, extracted from the F_v/F_m curve, depended on initial host density but did not depend considerably on experimental setup. Specifically, these times were: 14 days for initial concentration of 10^1 cells per mL, 11 days for 10^2 cells per mL, 8 days for 10^3 cells per mL, 5 days for 10^4 cells per mL, 2 days for 10^5 cells per mL, and 1 day for higher initial concentrations.

As shown in **Figure 3** and **Supplementary Figure 5**, the resulting behavior is qualitatively similar to that shown by the experiments, representing observations much more closely than the virulent model across the different initial conditions and treatments.

From a quantitative point of view, both the Akaike Information Criterion (AIC) [3] and the Mean Absolute Error (MAE, see Supplementary Materials description) [4] show that the phenomenological temperate model describes more closely than the virulent model the host dynamics we observed experimentally. Differences between AICs for the virulent model and the phenomenological temperate model for the three treatments with initial densities 10^1-10^4 cells per mL were larger than 25, indicating a far superior performance of the temperate model. An exponential transformation of this difference, $\exp(25 / 2) = 2.7 \cdot 10^5$ indicates that the temperate model is $2.7 \cdot 10^5$ times more probable to be the one that minimizes information loss in this case, making it dramatically preferred by this metric. In the preinfected treatment with an additional initial viral inoculation, for an initial density of 10^4 cells per mL the phenomenological temperate model has lower AICs by a difference of 19, corresponding to being $1.3 \cdot 10^4$ times more probable to minimize information loss relative to the virulent model. In cases with no viruses, and for the 10:1 and preinfected treatments with initial density of 10^5 cells per mL, the virulent model is preferred because it has one fewer parameter than the phenomenological temperate model. For the same initial density in the “preinfected plus additional virus” treatment, the temperate model is preferred but the associated probabilities are not meaningfully different (they are within a factor 5, although the correct choice of likelihood function is not clear). These conclusions are corroborated by the MAE results. For all but one of the experiments (the “preinfected plus additional virus” treatment with an initial density

of 10^5 cells per mL, where the virulent model gives different predictions than the temperate model), the residuals of the virulent model are larger, with a median difference of 2.15 (corresponding to a factor of 8.58) compared to the phenomenological temperate model.

Self-regulated temperate version: dynamic switch

The fact that the classic virulent model could not replicate our observed experimental data qualitatively, but the phenomenological temperate modification did replicate the behavior both qualitatively and quantiatively, evidenced the presence of an initial temperate mode for the virus. The fact that the main component differentiating both models is the physiology-informed timing for induction leads to the conclusion that both host density and physiology play a role in the timing of the viral switch to virulence.

Our data, however, did not provide enough information to understand (and therefore include in the model) the exact mechanisms that underlie induction. Thus, we further modified the temperate model to include mechanisms that could explain the timing at which viral pressure significantly decimates and causes declines in host populations. To this end, and based on our observations, we introduced several biologically-reasonable changes/assumptions that aimed at triggering induction through system self-regulation:

— The infection mode depends on the time that the virus spends in the extracellular *milieu* between lysis of prior hosts and finding and infecting the subsequent host (see main text). Specifically, we assumed that the infection is virulent if the typical time between infections is smaller than the average life span of the virus, measured as the typical viral decay rate.

Mathematically:

$$\frac{1}{k[H]} < \frac{1}{m_V}$$

or, equivalently, when:

$$[H] > \frac{m_V}{k} \tag{8}$$

— **Figure 2** shows that host autophagy co-occurs with induction. This suggests a link between the two that can be use to estimate induction rates. Those estimates need to take into account that **Figure 2** shows all cells in the population and not necessarily only infected cells. Thus, we further scaled the number of infected cells that undergo induction by the fraction of cells that show a temperate infection within the population. Our results were not qualitatively affected by this choice (and barely affected in a quantitative way, results not shown). Autophagy did not occur in the experiments (and therefore induction did not occur in the model) for the lowest initial density in the 10:1 treatment, and it was negligible for that same initial density in the preinfected treatments.

— If the infection is temperate, induction and associated viral replication and host lysis occurs after the infection leads to host stress (see main text). This is motivated by our observation that host autophagy occurs only in experiments where viruses are present (**Figure 2a**). Specifically, in this version of the model, induction is triggered when the following expression is *minimized*:

$$\frac{1}{\mu_I(t)} + \frac{1}{k[H]} = \frac{k[H] + \mu_I(t)}{\mu_I(t)k[H]} \tag{9}$$

From the point of view of the host, the expression is the result of adding the typical replication time of the infected host and time between infections. Thus, the expression considers the tradeoff between the need to replicate fast and the associated increase in infection risk (due to the consequent increase in the host density, which leads to higher encounter rates). From the point of view of the

virus, the expression signifies the overall generation time while temperate (or time between infections, as the replication of the infected cell always translates into “moving” to a new host). Importantly, this expression indirectly takes into account host stress in two ways: through density-dependent factors (self-shading, competition for resources) and other physiological factors including the temperate infection itself.

As mentioned above, we assumed that induction of intracellular temperate viruses occurs when the expression in Equation (9) transitions from decreasing to increasing.

Note that the condition given by Equation (8) is a sufficient condition for the infection to be virulent from the outset, whereas Equation (9) is a sufficient condition for induction. In consequence, infections that should be temperate will immediately after adsorption become virulent if the host is stressed enough for Equation (9) to be fulfilled. The host population typically surpasses the threshold for the infection mode condition (Equation (8)) before the induction condition (Equation (9)) occurs and, therefore, the newly-released viruses will ultimately result in virulent infections.

— Viral infectivity decreases fast out of the host, as shown by **Supplementary Figure 10a**. We implemented this condition during the initial temperate phase of the viral population, in which no new viruses are being produced and therefore viral aging can be easily tracked. Specifically, we imposed a higher mortality (or loss of infectivity) rate ($2d^{-1}$) for the extra-cellular viral population once the typical viral life span, $1/m_V$, is surpassed. In addition, to avoid an artificial recovery of this (old) viral population, we set a lower threshold of 10^{-1} cells per mL below which we set the population to zero.

As observed in **Supplementary Figure 5** and **Supplementry Table 3**, these data-inspired modifications provided results that are qualitatively and quantitatively close to those of the phenomenological temperate model. This similarity stems from emergent, self-regulated induction times that are close to our observed peaks in host physiological

stress for all treatments (shown indirectly in **Supplementary Figure 5** in the timing of population decline). From the quantitative point of view, because the curves obtained with the self-regulated model are similar to those of the phenomenological model, the AICs and ARs favor in most cases the simpler temperate model. Thus, the self-regulated version trades simplicity for generality. It requires more parameters but does not need from data-informed pre-set induction times and thus can potentially be used with any host-virus system.

d) Infected-host replication:

We assume that, while the virus is temperate, infected host cells can continue their usual life cycle and, therefore, continue replicating. Thus, the growth rate of infected hosts is implemented as:

$$\mu_I(t) = (1 - r_s)\mu(t) \tag{10}$$

that is, infected hosts replicate at the same growth rate as healthy hosts while the virus is temperate, and do not replicate at all when the virus is virulent. The justification for the latter is that, when virulent, the virus utilizes the synthesis machinery of the host, which prevents host replication.

As explained above, we assumed that infected-host replication produces new healthy hosts. In our simulations, distributing these replications between healthy and infected hosts, which is plausible if there is more than one virus per host opeing the possibility of superinfection, introduces some initial lag in the growth of the population. Because we lack information regarding the exact ratio of the population offspring that is represented by cells with complete (temperate) infective viruses, our choice here of assuming a complete healthy offspring is based on the best qualitative match with our experimental data.

e) Further considerations:

Other modeling choices

We computationally tested several options for each new mechanism we introduced in our models. For example, before we introduced temperateness, we tested whether the delay in the release of the virus could be explained by explicitly including the viral latent period in our virulent model. Although, for low initial densities, the decline in our experimental host populations occurs *several* latent periods after the experiments start, a potential accumulation of lags could be an alternative to temperateness when trying to explain our observations. To explicitly introduce the viral latent period, we tested a delayed version of the classic virulent model [5]. The resulting curves, however, show similar limitations to that of the classic virulent model (see **Supplementary Table 3**). For example, similarly to the classic virulent model this delayed version also fails to replicate the seemingly healthy-like host population growth that can be observed in the pre-infected treatments. This failure to represent our observations held even after introducing an additional modification by which viral traits depended explicitly on host physiology (specifically, burst size decreasing and latent period increasing as host growth rate decreases [6]). Another modification of the delayed model in which we decreased the efficiency of the virus by, *e.g.*, decreasing the contact rate (in the spirit of **Supplemental Figure 10b and c**) allowed the host population to grow in the simulated pre-infection treatment but only after a week-long delay, which contrasts with the apparently-healthy behavior of our experimental hosts from the outset. Thus, all these different versions of the delayed virulent model cannot explain qualitatively (nor quantitatively) the behavior observed in the laboratory.

For the temperate versions of the model, we also tested several viral trait value combinations, and several loss of infectivity expressions and thresholds, which only quantitatively affected the results. The final set of parameters chosen for **Figure 3** and **Supplementary Figure 5**, which were informed by our own experiments or the available literature, are biologically plausible and provided a behavior close to our observations.

As mentioned above, including vertical transmission by considering that the offspring of infected hosts can also result in new infected cells, introduced an additional delay in our simulations. Although we obtained the best results without vertical transmission, we

tested other healthy-to-infected offspring ratios, which did not significantly affect our results for the phenomenological version of the model; for the self-regulated version of the model, mid-to-large ratios led to deviations from observations for the preinfected treatments.

Finally, we considered removing the possibility of attachment by several viruses to the same host, which did not alter qualitatively our results. Removing superinfection entails the second term in Equation (5) with the simpler $k[H][V]$ wherein free viruses only attach and infect free hosts.

Extrapolation to intermediate initial densities

To extrapolate to initial densities beyond the ones we used for our experiments, we deduced phenomenological expressions that aimed to replicate how several of our parameters depended on the initial densities we sampled with the experiments.

For the timing at which the growth rate reaches its minimum value, t_μ , for example, we observed the following approximated behavior:

$$t_\mu = \begin{cases} -1.75\, log([Ehux]_0) + 25 \ \text{ if } \ [Ehux]_0 > 10^1 cells/mL \\ 19 \ \text{ otherwise} \end{cases} \tag{11}$$

in days, if the initial density expressed in cells per mL.

In addition, we deduced a phenomenological expression for t_s as a function of the initial host density:

$$t_s = \begin{cases} -1.3\, log([Ehux]_0) + 16 \ \text{ if } \ [Ehux]_0 < 10^4 cells/mL \\ 1 \text{ otherwise} \end{cases} \tag{12}$$

in days, with the initial concentration expressed in units of cells per mL. The expectation is that these times vary with different stress conditions.

Although, for the sake of generality, the results from **Figure 3** and **Supplementary Figure 5** were obtained using Eqs.(11)-(12) when relevant, using case-specific values for t_{μ}, or t_s does not alter our results qualitatively.

Supplementary Note 2: Revisiting the rules of infection: Description of the infection according to our best model

As mentioned above, our data show conclusively the existence of an initial temperate mode for the virus, and offer important information about the factors influencing such mode. Importantly, our models provide further information that can help reveal the mechanisms underlying the timing of viral induction. Thus, the closeness of the temperate models to our observations allows us to hypothesize which factors may be important for this induction:

— The virus is necessarily “undecided” from the beginning.

— The traveling time to reach the host decides whether the infection is temperate or virulent (Equation 8).

— A combination of host density and physiology triggers induction (either caused by the host alone or facilitated by the virus as well), and the timing is close to that provided by the condition encoded in Equation (9).

— Given the conditions above (and their timing), the switch is effectively unidirectional, going from temperate to virulent but not *vice versa*.

— Infected cells show more stress than healthy cells, and healthy hosts show more stress in a virus-filled medium than when cultured without viruses, reason why autophagy is seen only in experiments in which viruses are present. The fact that both preinfection treatments show similar host dynamics reinforces this idea.

Future work aims to better understand these potential mechanisms (from both ecological and evolutionary perspectives), and test the hypotheses above in the laboratory.

Supplementary References

1. Monod, J. La technique de culture continue: theorie et applications. (1950).
2. Mackinder, L. C. M. *et al.* A unicellular algal virus, Emiliania huxleyi virus 86, exploits an animal-like infection strategy. *J. Gen. Virol.* **90**, 2306–2316 (2009).
3. Akaike, H. A new look at the statistical model identification. *IEEE Trans. Automat. Contr.* **19**, 716–723 (1974).
4. Lilliefors, H. W. On the Kolmogorov-Smirnov test for normality with mean and variance unknown. *J. Am. Stat. Assoc.* **62**, 399–402 (1967).
5. Bonachela, J. A. & Levin, S. A. Evolutionary comparison between viral lysis rate and latent period. *J. Theor. Biol.* **345**, 32–42 (2014).
6. Choua, M. & Bonachela, J. A. Ecological and evolutionary consequences of viral plasticity. *Am. Nat.* **193**, 346–358 (2019).
7. Nissimov, J. I. *et al.* Biochemical diversity of glycosphingolipid biosynthesis as a driver of Coccolithovirus competitive ecology. *Environ. Microbiol.* **21**, 2182–2197 (2019).
8. Balch, W. M., Kilpatrick, K. A., Holligan, P., Harbour, D. & Fernandez, E. The 1991 coccolithophore bloom in the central North Atlantic. 2. Relating optics to coccolith concentration. *Limnol. Oceanogr.* **41**, 1684–1696 (1996).
9. Laber, C. P. *et al.* Coccolithovirus facilitation of carbon export in the North Atlantic. *Nat. Microbiol* **3**, 537–547 (2018).

Supplementary Table 1: Summary of independent laboratory experiments, initial host densities, diagnostic stains, and treatments (media type and infection regime) conducted in this study (MOI is Multiplicity of Infection).

Experiment	Days Sampled	Media		Uninfected					10:1 MOI Co-incubation					Pre-infected					Pre-infected & Co-incubation					Format
		f/2	SW	10^5	10^4	10^3	10^2	10^1	10^5	10^4	10^3	10^2	10^1	10^5	10^4	10^3	10^2	10^1	10^5	10^4	10^3	10^2	10^1	
I	1, 2, 3, 4, 5	✔		✔	✔	✔	✔	✔	✔	✔	✔	✔	✔	✔	✔	✔	✔	✔	✔	✔	✔	✔	✔	Culture Flasks
II	1, 2, 3, 4, 5, 7	✔	✔	✔	✔	✔	✔		✔	✔	✔	✔												96-Well Plates
III	1, 3, 5	✔		✔	✔	✔	✔	✔	✔	✔	✔	✔	✔											6-Well Plates
IV[1]	0, 1, 3, 6, 9, 12	✔	✔				✔					✔					✔							Culture Flasks
V[2]	0, 1, 2, 3, 5, 8, 13, 21	✔	✔*	✔	✔	✔	✔	✔	✔	✔	✔	✔	✔	✔	✔	✔	✔	✔	✔	✔	✔	✔	✔	Culture Flasks
VI[3]	0, 1, 2, 3, 5, 7, 9, 11, 14	✔	✔		✔	✔	✔			✔	✔	✔			✔	✔	✔							Culture Flasks
VII[4]	2, 3, 4, 5, 6, 7	✔		✔	✔	✔	✔	✔	✔	✔	✔	✔	✔											Culture Flasks

* 10^2 Initial host density treatment only.
[1] Diagnostic metrics: SYTOX, Lysotracker, UV autofluorescence, F_v/F_m.
[2] Diagnostic metrics: SYTOX, Lysotracker. Data from this experiment are shown in **Supplementary Figure 4**.
[3] Diagnostic metrics: SYTOX, UV autofluorescence, F_v/F_m. Data from this experiment are shown in **Figure 4a**.
[4] Diagnostic metrics: SYTOX

Supplementary Table 2: Parameters used in virulence and temperate theoretical models. Symbols, units, and parametrization used with the different versions of the model are listed along with sources.

Symbol	*Definition*	*Units*	*Value*	*Source*
t	Time	*days*	Variable	—
$[H]$	Concentration healthy hosts	$cells \cdot L^{-1}$	Variable	—
$[I]$	Concentration infected hosts	$cells \cdot L^{-1}$	Variable	—
$[V]$	Concentration free viruses	$viruses \cdot L^{-1}$	Variable	—
μ	Growth rate, healthy hosts	$days^{-1}$	Variable	—
μ_I	Growth rate, infected hosts	$days^{-1}$	Variable	—
μ_{max}	Maximum growth rate	$days^{-1}$	1.0 (for f/2), 0.75 (for SW)	Experiments
μ_{min}	Minimum growth rate	$days^{-1}$	0.75 (0.70 if initial density 10^4 $cells \cdot L^{-1}$)	Experiments
m	Host mortality rate	$days^{-1}$	0.0	Experiments
t_μ	Time at which $\mu=\mu_{\mathrm{min}}$	*days*	19, 17, 14, 9, 5 (for initial densities 10^4, 10^5, 10^6, 10^7, 10^8 $cells \cdot L^{-1}$, resp.); 2 if initial density larger than 10^8 $cells \cdot L^{-1}$	Experiments
K	Carrying capacity	$cells \cdot L^{-1}$	$6.5 \cdot 10^9$ (for f/2); $5 \cdot 10^6$,$4 \cdot 10^7$,$3 \cdot 10^8$ (for SW with initial densities 10^5, 10^6, 10^7 $cells \cdot L^{-1}$, resp.)	Experiments
B	Viral burst size	$viruses \cdot host^{-1}$	100	Nissimov et al 2019 [7]
k_L	Viral lysis rate	$days^{-1}$	0.5	Nissimov et al 2019 [7]
k	Adsorption rate	$L \cdot days^{-1}$	$1.44 \cdot 10^{-8}$	Nissimov et al 2019 [7]
m_V	Viral decay rate	$days^{-1}$	1/3	Experiments; Supplementary Figure 9a
t_s	Switching time	*days*	14, 11, 8, 5, 2 (for initial densities 10^4, 10^5, 10^6, 10^7, 10^8 $cells \cdot L^{-1}$, resp.); 1 if initial density larger than 10^8 $cells \cdot L^{-1}$	Experiments
r_s	Switch temperate/lytic	—	0 or 1 (see text)	—

Supplementary Table 3: Summary and comparison of features from virulence and temperate models. Empirical phenomena captured by each of the virulence and temperate theoretical models (MOI is Multiplicity of Infection).

Virus<br>Feature	Lytic-only (*i.e.*,virulent)	Temperate,<br>data-informed induction time	Temperate,<br>self-regulated induction time
Decline time dependent on initial density for 10:1 MOI treatment	YES	YES	YES
Decline time dependent on initial density for pre-infected treatments	NO	YES	YES
Undetectable initial host mortality	NO	YES	YES
Lower host density at collapse for seawater media	YES	YES	YES

Supplementary Figures

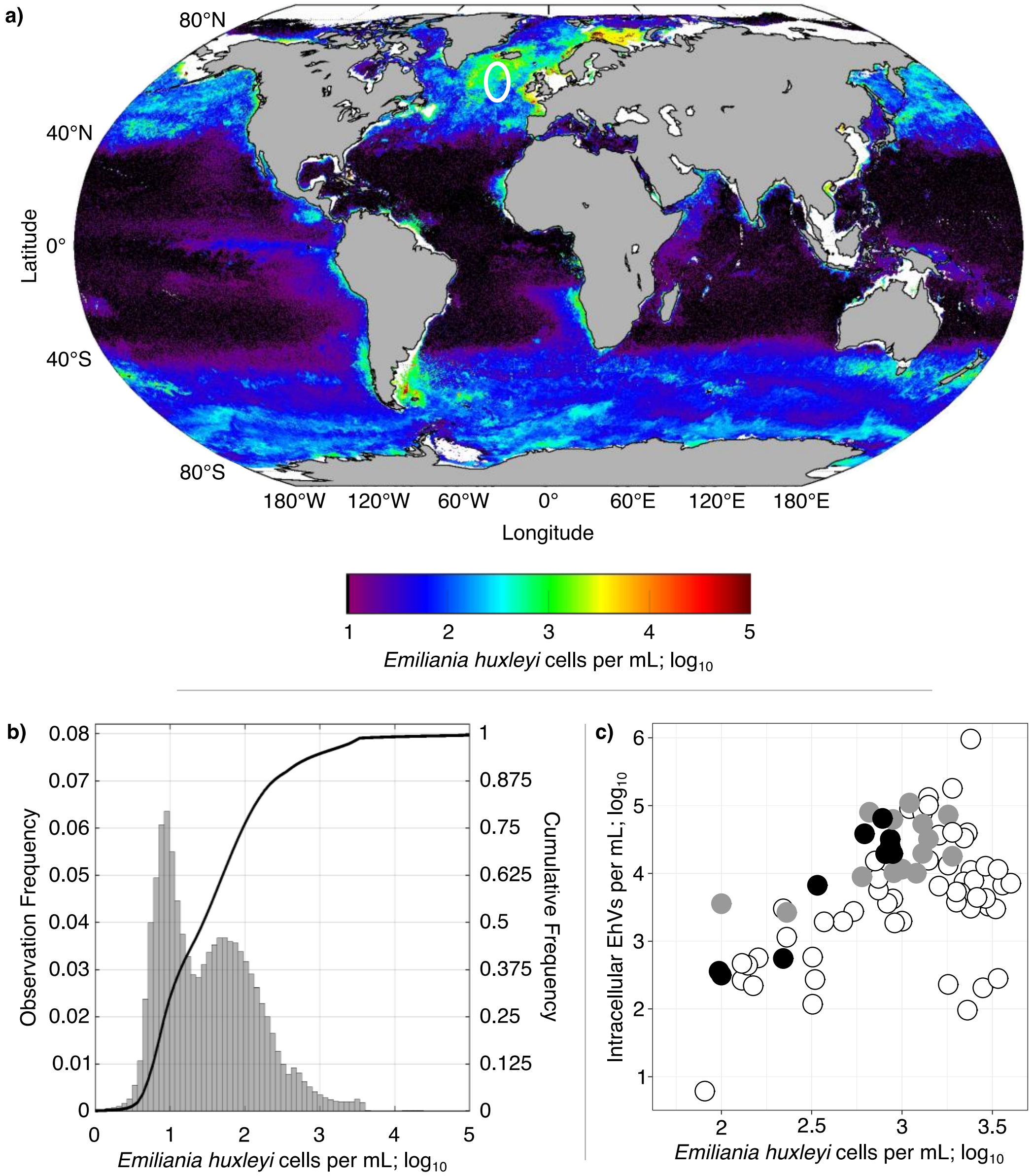

Supplementary Figure 1: **The spatial and density distributions of *Emiliania huxleyi* in the global ocean where *E. huxleyi* densities rarely exceed 10^3 cells per mL.** The maximum *E. huxleyi* density observed between 2003 and 2017 in any given pixel (pixelwise maxima; ~ 9 km by 9 km pixels; water column depths > 100 m) are shown as **(a)** geographic distribution and as **(b)** a frequency distribution. *E. huxleyi* densities were estimated from ocean color satellite retrievals of Particulate Inorganic Carbon (PIC) and empirical relationships derived from *in situ* bloom measurements [8]. Natural host and intracellular virus densities from all depths across stations characterized as early (white points), late (grey points), and post (black points) viral infection **(c)** are shown from the NA-VICE cruise undertaken in June - July 2012 in the Northeast Atlantic (see white circle in panel **a;** [9]). See **Figure 1** inset (top left panel) for comparison to laboratory studies.

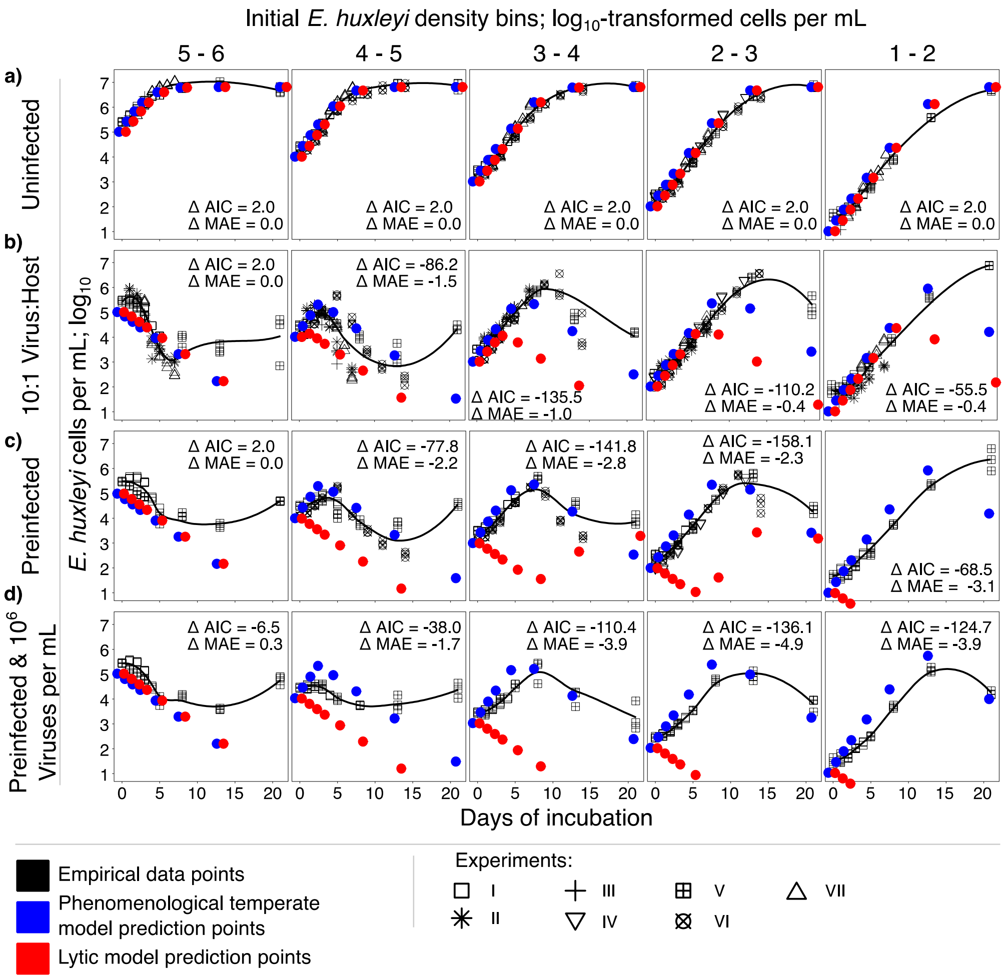

Supplementary Figure 2: **Empirical *versus* virulence and phenomenological temperate theoretical model data points.** Empirical data (black) and virulence (red) and phenomenological temperate (blue) model prediction points for **(a)** uninfected, **(b)** 10:1 virus:host Multiplicity of Infection (MOI) co-incubations, **(c)** pre-infected, and **(d)** pre-infected with added viruses; all initial host density treatments are shown independently and are indicated on the top of each panel column (log range). Modeled data points in **(c)** and **(d)** assume a pre-infection rate of ~ 99% as predicted by the model after 2 h co-incubation of 10^6 hosts and 10^7 viruses per mL. Data points are shaped according to experiment (**Supplementary Table 1**). Empirical data are shown with LOESS lines of best fit (solid black lines). To be consistent with the empirical data, modeled prediction points (circles) from days 0, 1, 2, 3, 5, 8, 13, and 21 are shown. Difference in Akaike Information Criterion (i.e, Δ AIC; $AIC_{phenomenological\ temperate}$ - $AIC_{virulent}$) and Mean Absolute Error (Δ MAE; log_{10}; $MAE_{phenomenological\ temperate}$ - $MAE_{virulent}$) are shown, where negative values show the phenomenological temperate model fitting empirical data (**Figure 1a**) better than the virulent model despite being penalized for having extra parameters. Source data are provided at https://github.com/benjaminwilliamknowles/Coup-de-Grace.

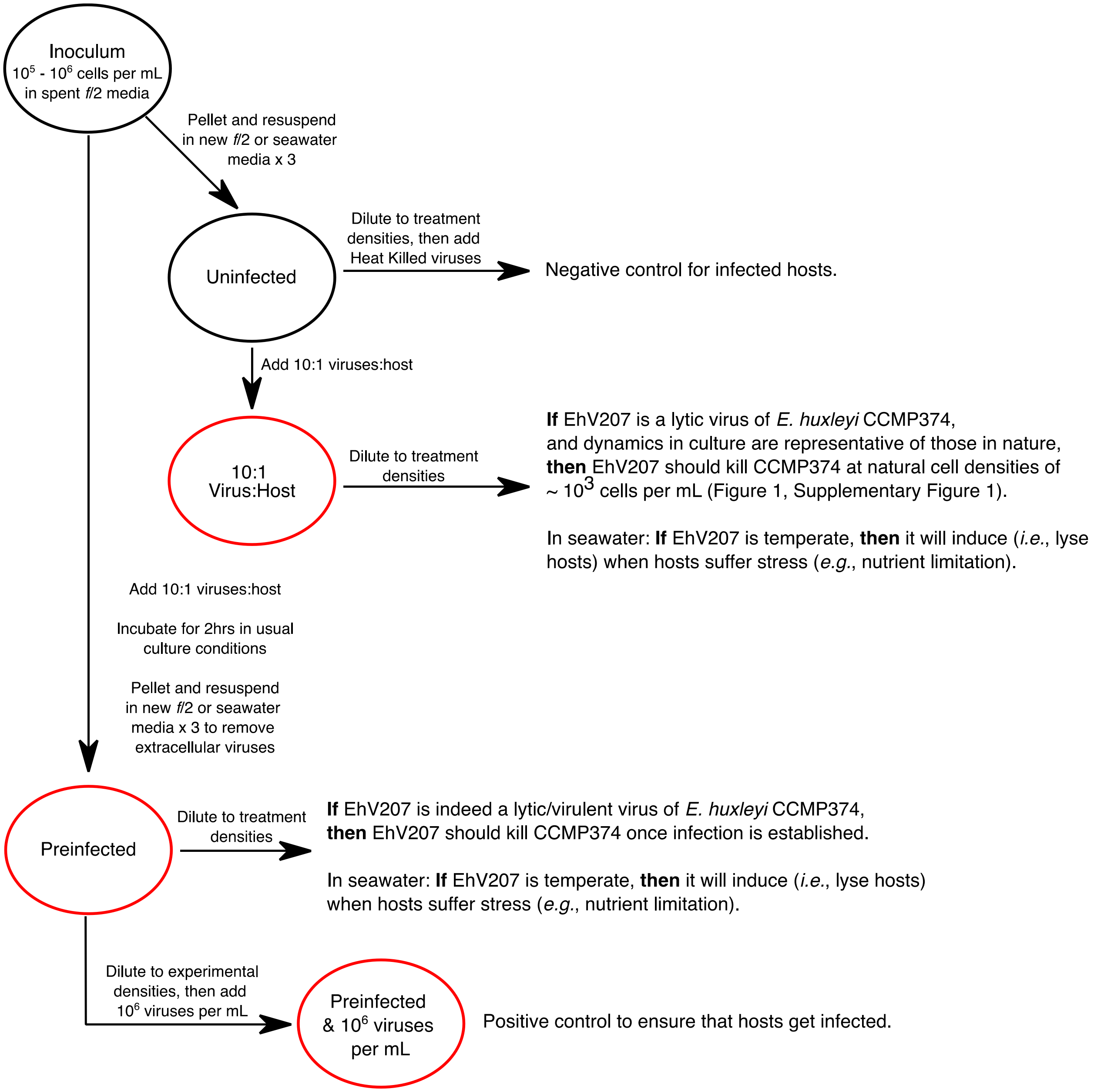

Supplementary Figure 3: **Overview of experimental treatments and rationale.** Experimental processes are shown by labeled lines, cultures that had either been infected (red ellipses) or not (black ellipses), and rationale shown as if/then statements.

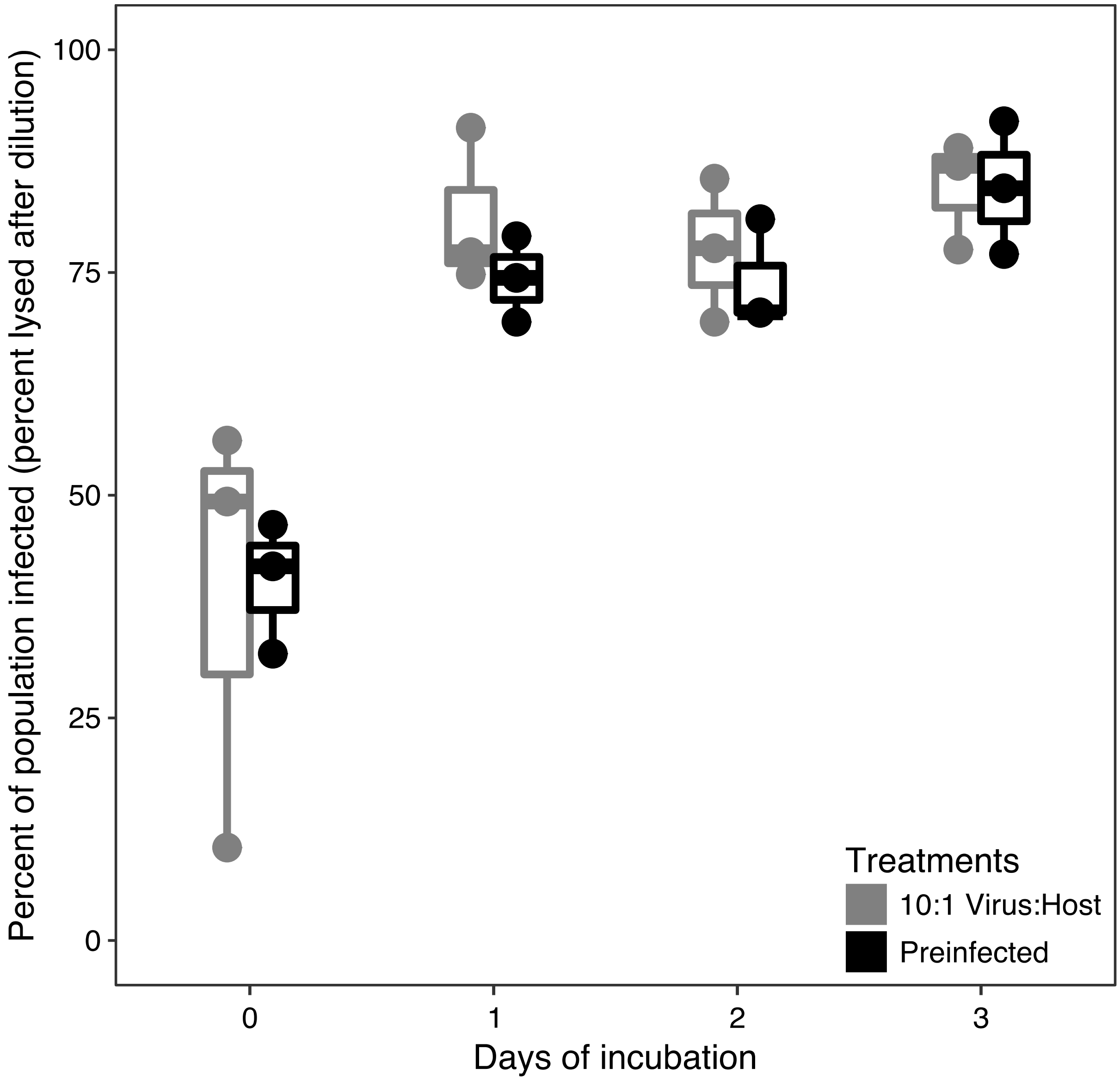

Supplementary Figure 4: The frequency of infected cells over the first three days of incubation. Triplicate pre-infected (black points and box and whiskers) and 10:1 virus:host Multiplicty of Infection (MOI) co-incubations (grey points and box and whiskers) are shown from t_0 samples in *f*/2 media in a representative experiment (Experiment V; **Supplementary Table 1**). Whiskers range from minimum to maximum values (*i.e.*, 0th – 100th percentile), boxes show mean as horizontal bars (50th percentile) and box shoulders show 25th and 75th percentile as lower and upper shoulders, respectively. Source data are provided at https://github.com/benjaminwilliamknowles/Coup-de-Grace.

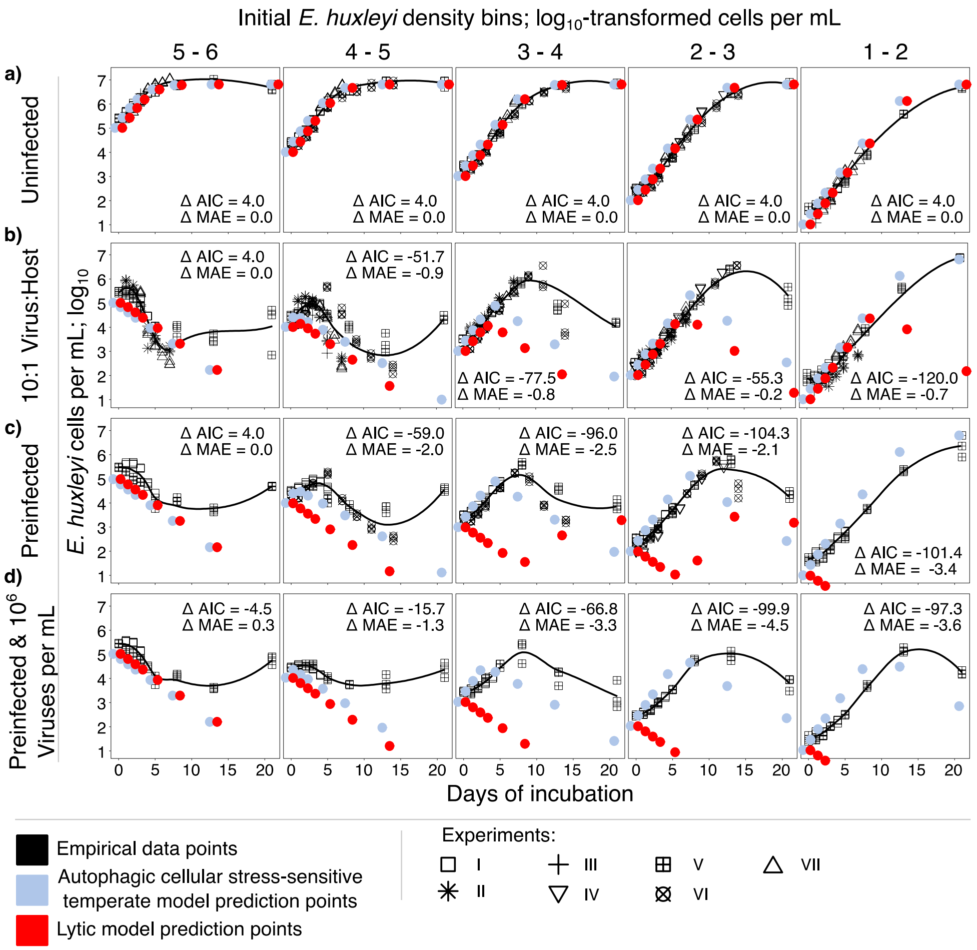

Supplementary Figure 5: **Empirical *versus* virulent and self-regulated, autophagy-triggered induction temperate theoretical model data points.** Comparisons between empirical data (black) and virulent (red) and self-regulated autophagy-triggered induction temperate virus model (light blue) predictions for **(a)** uninfected, **(b)** 10:1 virus:host Multiplicity of Infection (MOI) co-incubations, **(c)** pre-infected, and **(d)** pre-infected with added viruses. All initial density treatments are shown independently and are indicated on the top of each panel column ($\log_{10}$ range). Modeled data points in **(c)** and **(d)** show a pre-infection rate of ~99 % as predicted by the model after 2 h co-incubation of 10^6 hosts and 10^7 viruses per mL. Data points are shaped by experiment (see inset symbol legend and **Supplementary Table 1**). Empirical data are shown with LOESS lines of best fit (solid black lines). To be consistent with the empirical data, modeled prediction points (circles) from days 0, 1, 2, 3, 5, 8, 13, and 21 are shown. Note that prediction points and LOESS lines for the cell stress-sensitive temperate virus model (light blue) are offset slightly to the left and virulent model predictions (red) offset to the right to avoid overplotting. Difference in Akaike Information Criterion (i.e, Δ AIC; $AIC_{\text{autophagy-triggered temperate}}$ - AIC_{virulent}) and Mean Absolute Error (Δ MAE; $\log_{10}$; $MAE_{\text{autophagy-triggered temperate}}$ - MAE_{virulent}) are shown, where negative values show the self-regulated autophagy-triggered induction temperate model fitting empirical data (**Figure 1a**) better than the virulent model despite being penalized for having extra model terms. Source data are provided at https://github.com/benjaminwilliamknowles/Coup-de-Grace.

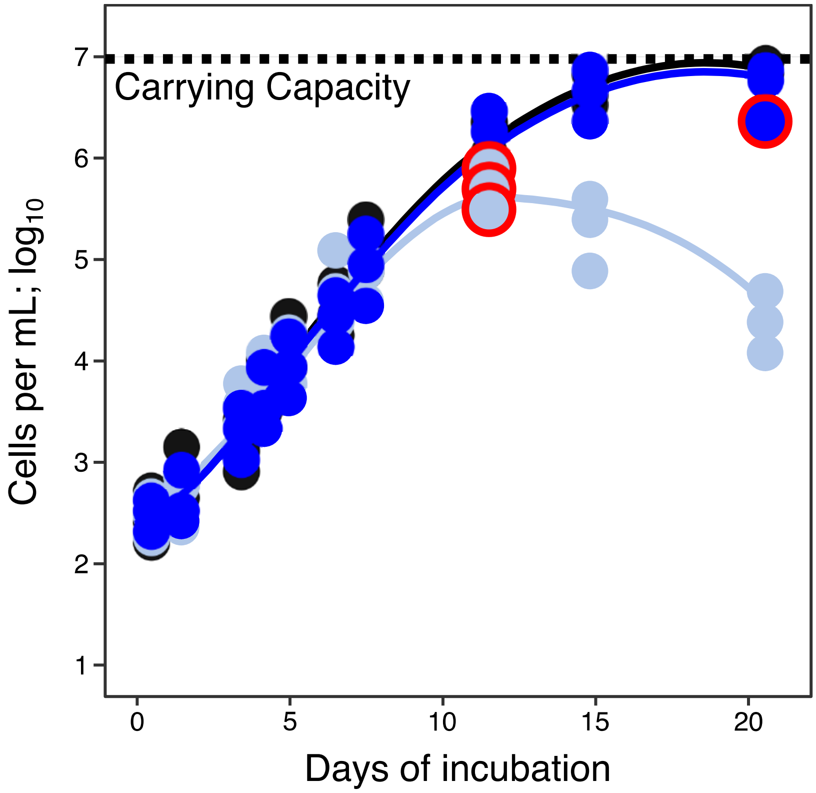

Supplementary Figure 6: Schematic of how 'carrying capacity' and 'lytic density' parameters were extracted from host growth dynamics. Carrying capacity (dashed line) was estimated as the average maximum host density observed in uninfected cultures (black dots and LOESS line) that reached stationary phase. For *f*/2 rich media experiments, this value was approximately 10^7 cells per mL. For seawater experiments, carrying capacity varied by treatment and experiment. Blue dots and LOESS lines show two different scenarios under which lytic density was estimated. The light blue points and line show a scenario where all replicates were suppressed by lysis. In this case, the lytic density was estimated as the maximum host density attained in each replicate (light blue points with red rings). The dark blue points and line show a scenario where not all replicates show viral-mediated decline. In this scenario, the density at which cultures visibly separated 'downward' from other replicates, after having previously tracked with those replicates, was used as the lytic density (dark blue point with red ring). Note that because cultures were not sampled daily, lysis may have been initiated between sampling points and lytic densities may exceed these estimates.

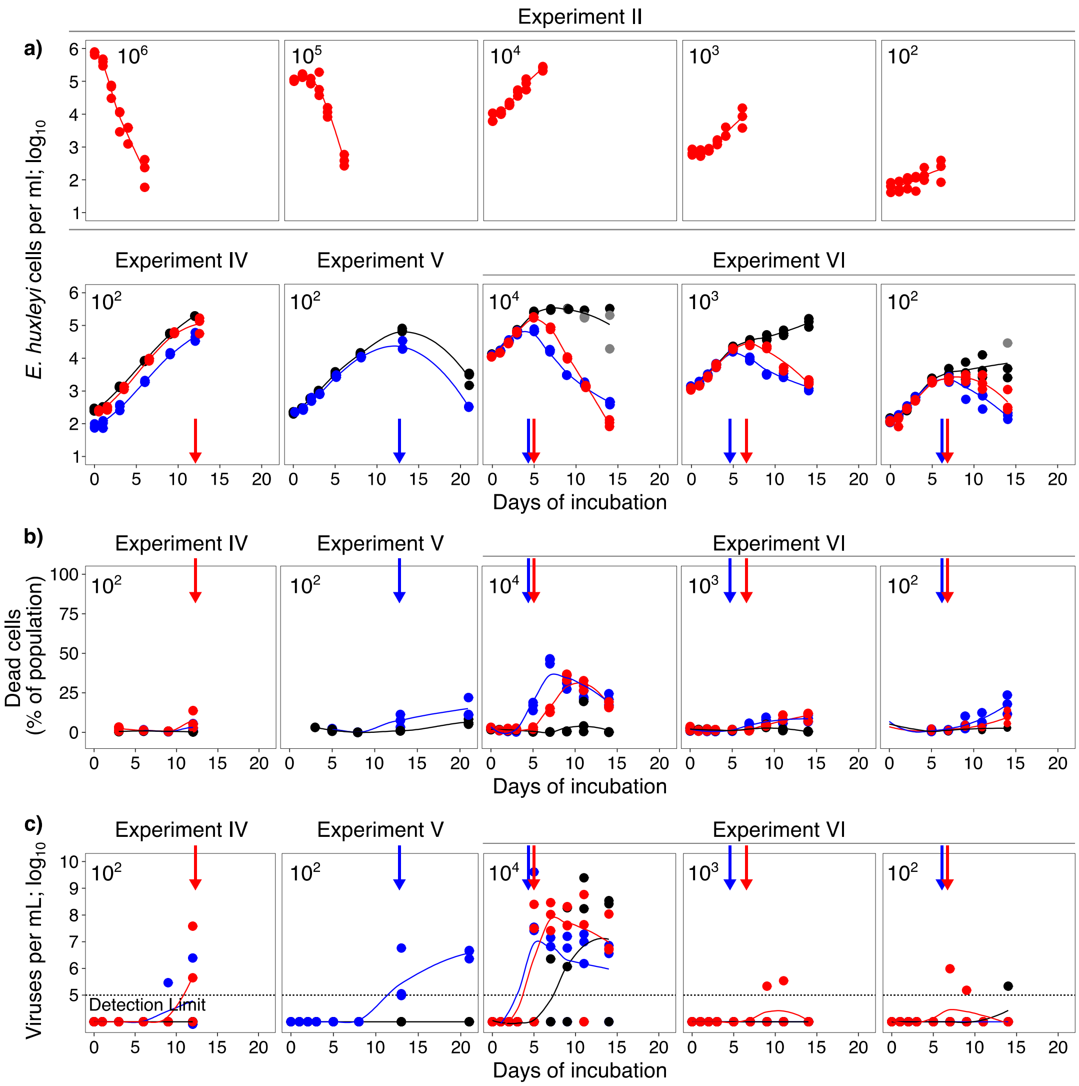

Supplementary Figure 7: Host density, death and extracellular virus densities over time in seawater incubations. (a) Host densities ($\log_{10}$-transformed; n = 427 independent counts across 4 independent experiments), **(b)** proportion of dead host cells (n = 337 independent counts across 4 independent experiments; SYTOX stain positive, data points with > 10^3 cells per mL initial threshold), and **(c)** extracellular viral densities ($\log_{10}$-transformed; n = 337 independent counts across 4 independent experiments) in populations of uninfected hosts (black points and LOESS lines), infected 10:1 virus:host Multiplicity of Infection (MOI) co-incubations (red points and LOESS lines), and pre-infected hosts without extracellular viruses (blue points and LOESS lines) across a range of initial host densities from 10^6 to 10^2 cells per mL from Experiments II (top row only), IV, V, and VI (see **Supplementary Table 1**). Data in **(a)** and **(b)** were generated by flow cytometry; **(c)** by qPCR (see **Supplementary Figure 12** for standard curves and thresholds). Vertical arrows show average onset of lysis in each treatment. Independent experiments are summarized in Supplementary Table 1 and shown individually here; see panel titles. Source data are provided at https://github.com/benjaminwilliamknowles/Coup-de-Grace.

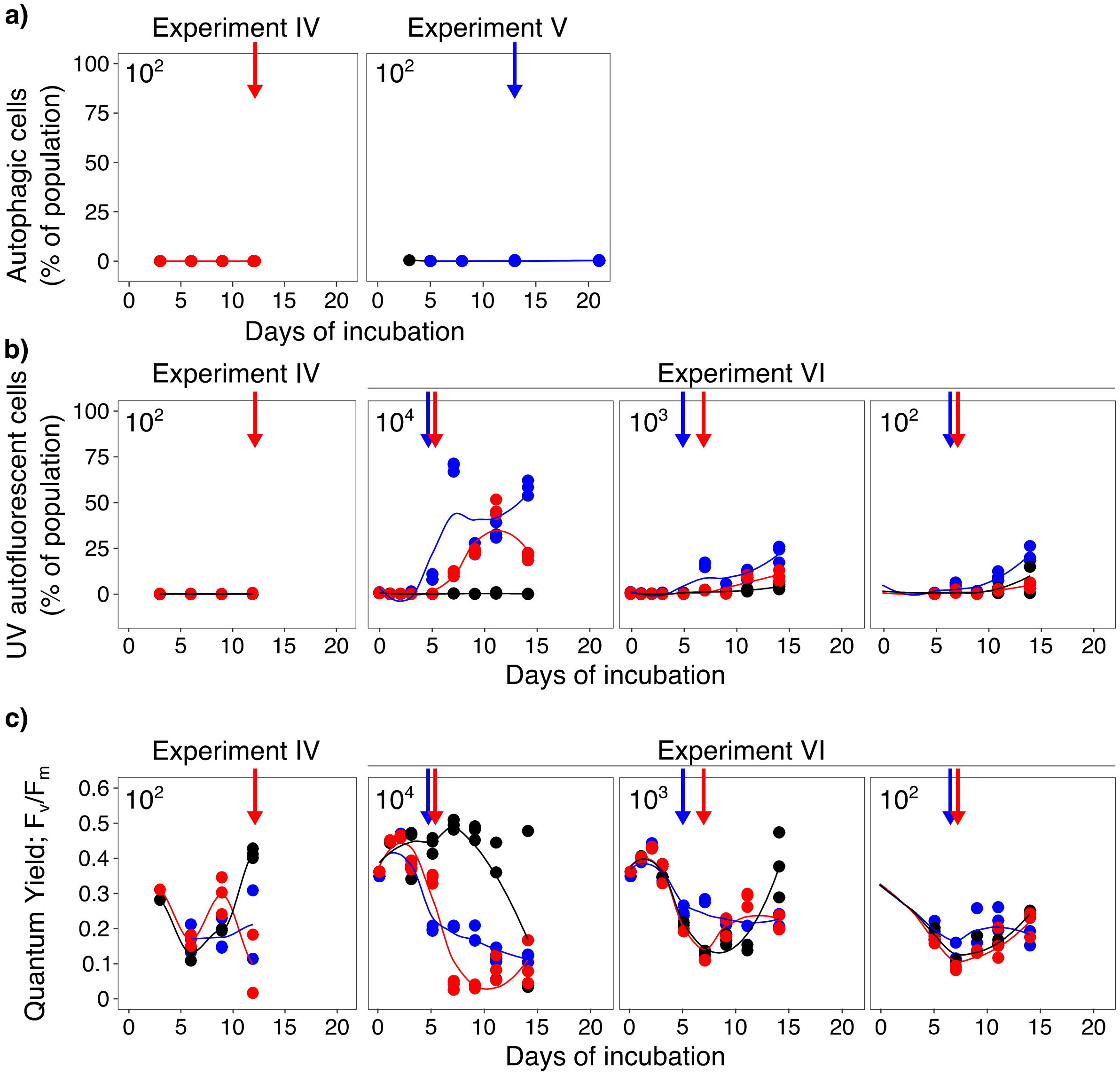

Supplementary Figure 8: **Physiological markers in seawater incubations. (a)** Percent of the population undergoing autophagy (n = 102 independent counts across 2 independent experiments), **(b)** showing elevated UV autofluorescence from metabolic dysfunction (n = 289 independent counts across 2 independent experiments), or **(c)** declining photochemical quantum yield of PSII (F_v/F_m; n = 269 independent counts across 2 independent experiments), in populations of uninfected hosts (black points and LOESS lines), infected 10:1 virus:host Multiplicity of Infection (MOI) co-incubations (red points and LOESS lines), and pre-infected hosts without extracellular viruses (blue points and LOESS lines) across a range of initial host densities from 10^6 to 10^2 cells per mL from Experiments IV, V, and VI (see **Supplementary Table 1**). All data points have a > 10^3 cells per mL initial threshold (**Supplementary Figure 12**). Vertical arrows show average onset of lysis in each treatment. Independent experiments are summarized in Supplementary Table 1 and shown individually here; see panel titles. Source data are provided at https://github.com/benjaminwilliamknowles/Coup-de-Grace.

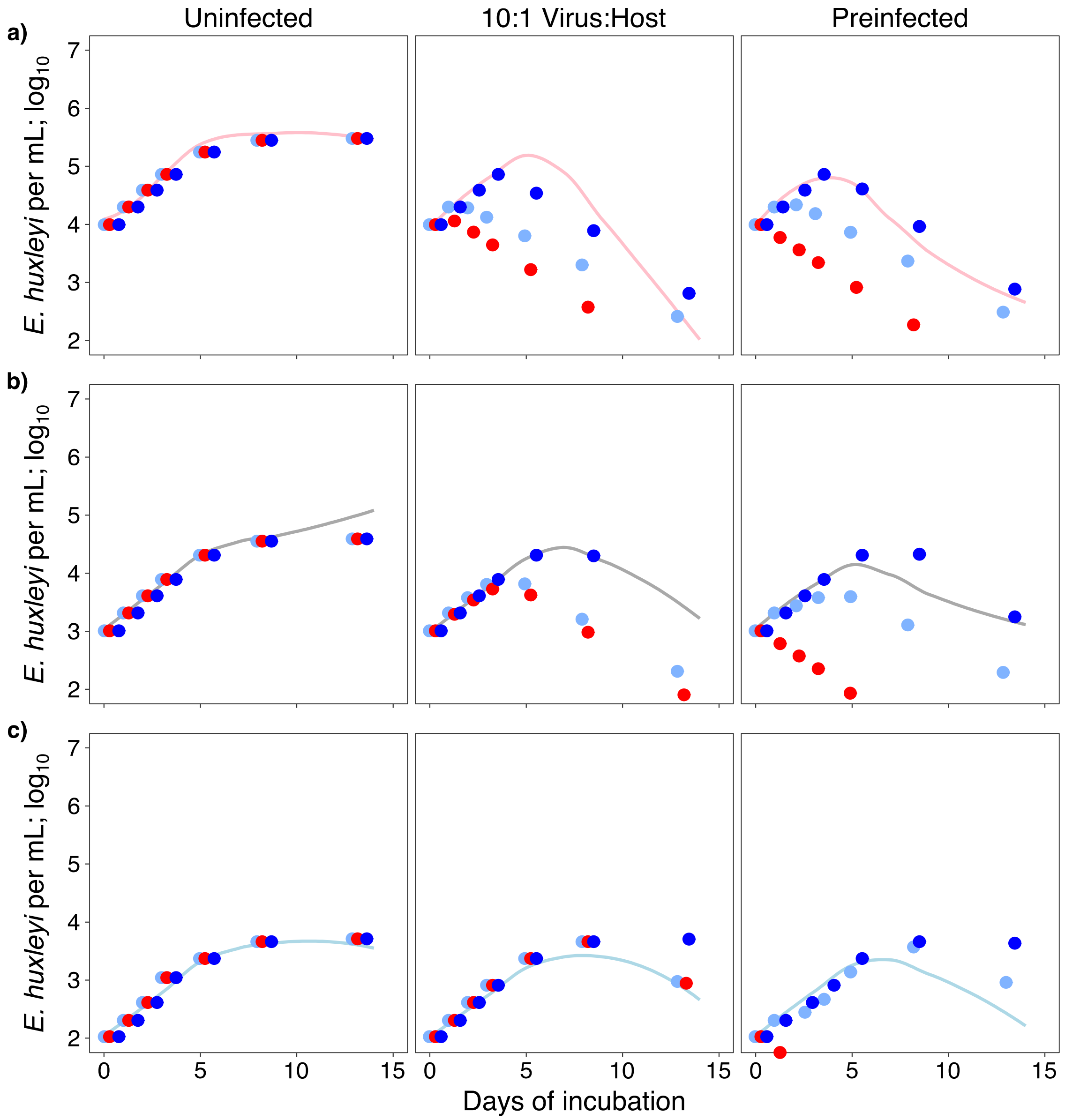

Supplementary Figure 9: Empirical *versus* virulence and temperate theoretical model data points in seawater incubations shown in Figure 4a-c. Comparisons between empirical data (LOESS lines of best fit from **Figure 4a** colored by initial host density) and virulence (red circles) and both phenomenological temperate virus model (dark blue circles) and self-regulated temperate virus model (light blue circles) predictions for cultures with initial host densities of **(a)** 10^4, **(b)** 10^3, **(c)** 10^2 cells per mL. Modeled data points in the Preinfected treatment show a pre-infection rate of ~ 99 % as predicted by the model after 2 h co-incubation of 10^6 hosts and 10^7 viruses per mL. To be consistent with the empirical data, modeled prediction points (circles) from days 0, 1, 2, 3, 5, 8, 13 are shown. Note that prediction points from each model are offset slightly to avoid over-plotting. Source data are provided at https://github.com/benjaminwilliamknowles/Coup-de-Grace.

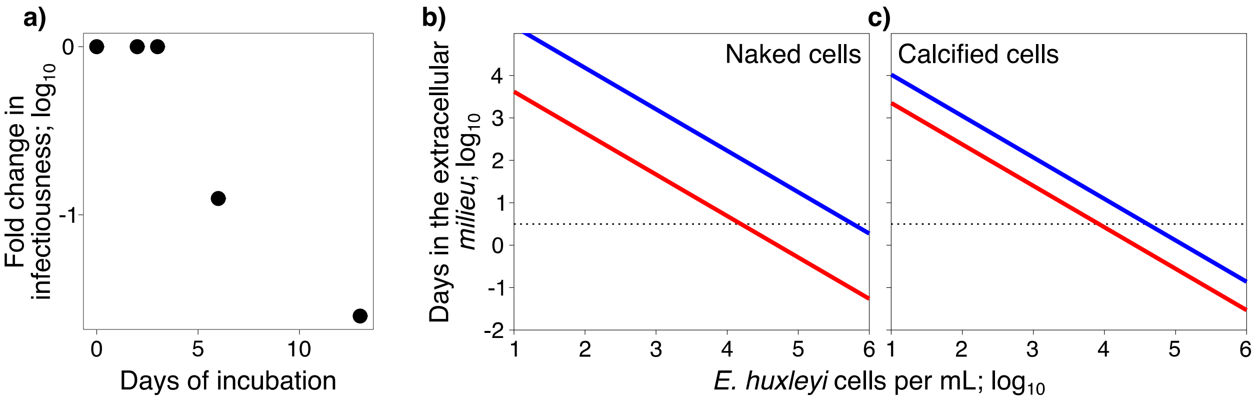

Supplementary Figure 10: **Timescales of viral loss of infectiousness and residence in the extracellular environment between lysis and subsequent infection.** The *Coccolithovirus* EhV207 experiences appreciable declines in infectiousness in seawater on the order of ~ 3 days (**a**; dashed horizontal line in **b** and **c**). Viruses not encountering a host within that period very likely lose infectiousness before they can infect a host. Predicted time an EhV virus spends in the extracellular environment before contacting a **(b)** naked or **(c)** calcified *E. huxleyi* cell as a function of host density under calm (dissipation rate: 10^{-8} m^2 s^{-3}; blue lines) or turbulent (stormy; dissipation rate: 10^{-4} m^2 s^{-3}; red lines) environmental conditions. Simulations suggest that viruses are only able to encounter a host within ~ 3 days if hosts are at ~ 10^4 or 10^5 cells per mL (*i.e.*, where the dashed lines cross the blue and red lines in **b** and **c**) regardless of turbulence regime or calcification state. Given that natural densities are appreciably less than this, it is predicted that infection cannot successfully propagate *via* virulent transmission. Source data are provided at https://github.com/benjaminwilliamknowles/Coup-de-Grace.

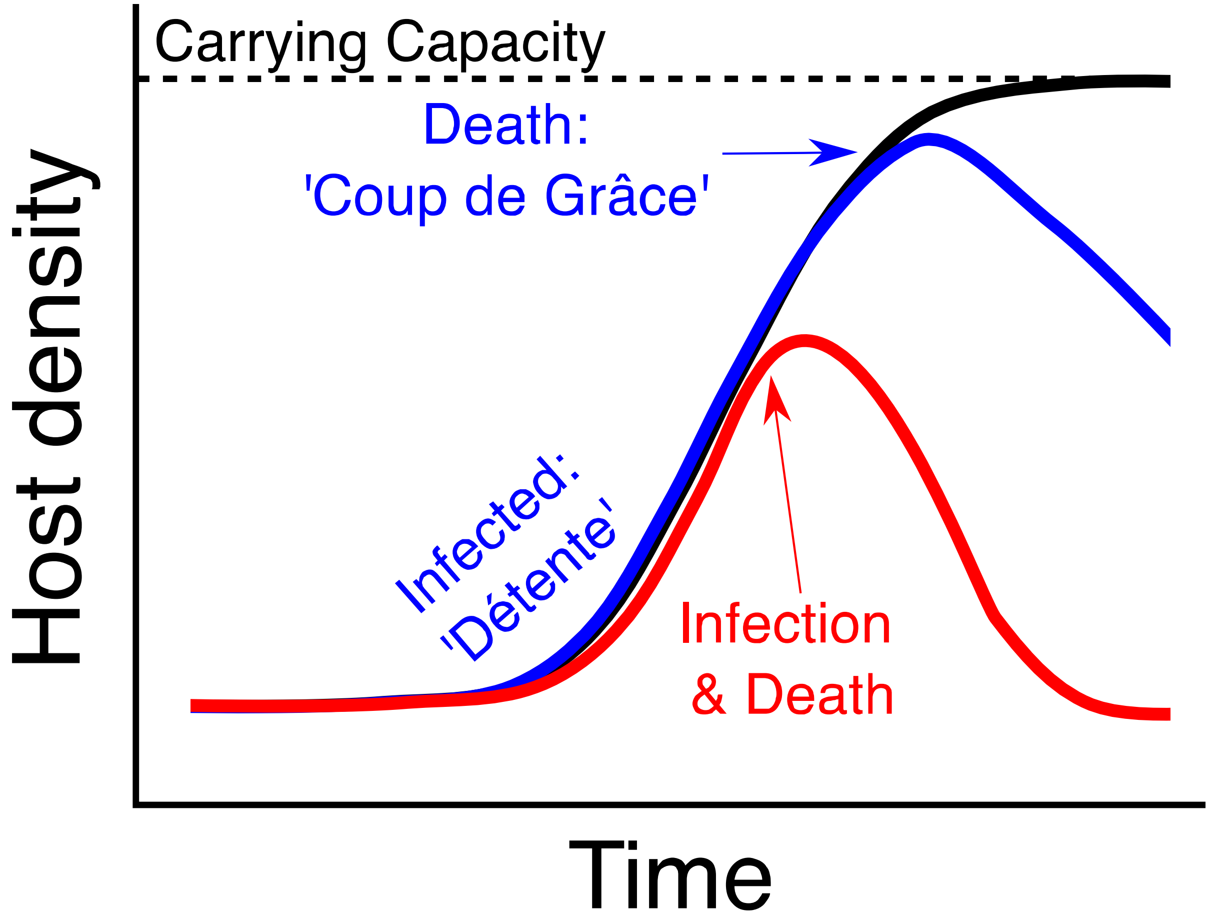

Supplementary Figure 11: **Temperate *versus* virulence dynamics, compared against a hypothetical uninfected control.** The virulent model (red line and text) intimately couples infection – the outcome of rising host densities – and lysis. In contrast, hosts may be infected at any time in the temperate model (blue line and text). A period of asymptomatic infection (virus-host *'Détente'*) exists up until host growth peaks from a process like nutrient limitation, at which time the temperate viruses induce and lyse the stressed hosts (the viral-mediated *'Coup de grâce'*). Note that carrying capacity (black dashed horizontal line), where uninfected populations (black line and text) would suffer bottom-up restriction of growth, is generally unable to be measured in environmental systems due to the presence of predators, competition, *etc.*, and may only be measurable in laboratory cultures. However, experimental elevation of carrying capacity in the environment (*e.g.*, by adding nutrients to nutrient limited systems) may present a means to determine if viruses are following a temperate or virulent strategy. Virulent killing thresholds will be insensitive to nutrient addition, while temperate killing thresholds will vary as a function of nutrient addition.

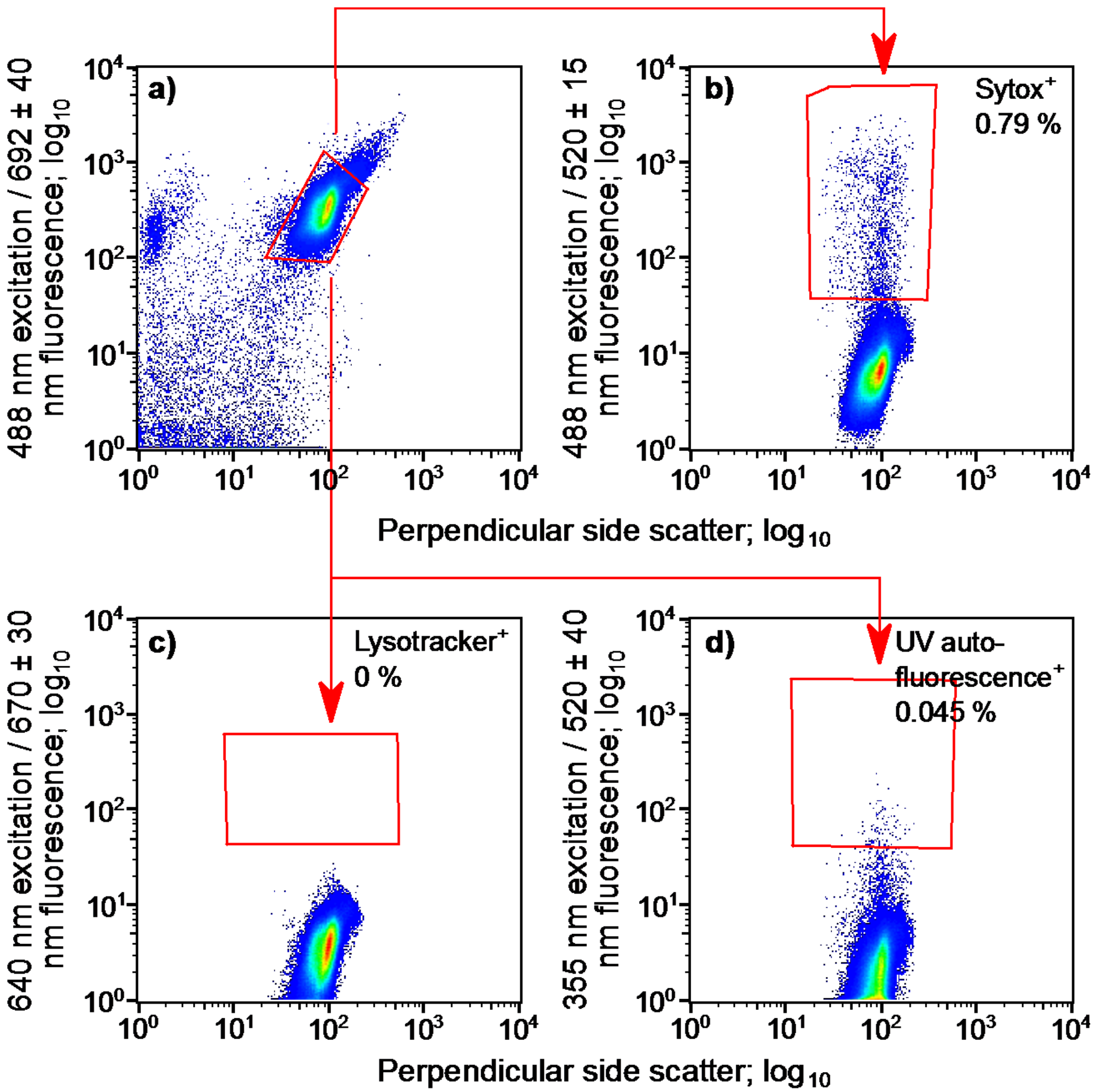

Supplementary Figure 12: Schematic summary of flow cytometry gating. (**a**) Intact *Emiliania huxelyi* cells were identified and counted *via* chlorophyll fluorescence (488 nm excitation / 692 ± 40 nm fluorescence; $\log_{10}$) and event size (perpendicular side scatter; $\log_{10}$) gates. Intact cells identified using this gate (red lines and arrows) were then analysed using excitation/fluorescence wavelengths to discern whether cells were positive or negative for the diagnostic markers (**b**) Sytox (a live/dead stain), (**c**) Lysotracker (to measure autophagy), and (**d**) UV autofluorescence (a novel metric likely associated with cellular redox stress). Experiments differed in which markers were used (see **Supplementary Table 1** for details). Gates (red boxes) were drawn to minimize false positive signals, allowing ≤ 1 % of event to be counted as positive in negative controls, as shown in the representative sample here. Panels **b-d** show the percentage of events in each cytogram falling into the gates (*i.e.*, false positive rate).

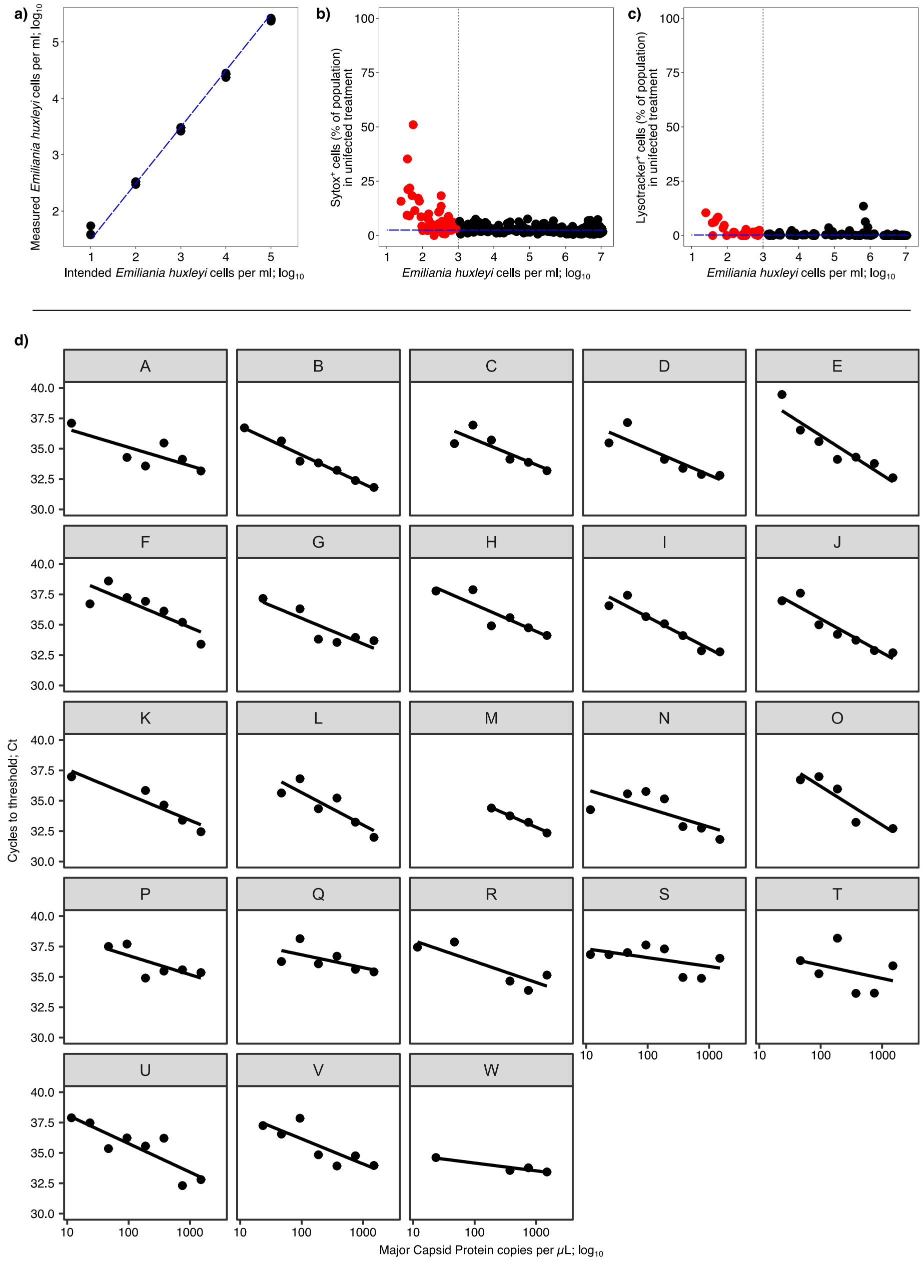

Supplementary Figure 13: **Detection limits for physiological assays and viral quantification.** (**a**) *Emiliania huxleyi* density, (**b**) the percentage of dead cells, and (**c**) the percentage of cells undergoing autophagy in the uninfected treatment t_0 samples. Given control cells are uninfected, they are expected to show similar basal levels of death (~5% of population; where blue dashed line would cross the y-axis in **b**) and autophagy (~1 % of population; where blue dashed line would cross the y-axis in **c**), allowing the detection limit (*i.e.*, the host density at which false positives attenuate) to be set at 10^3 cells per mL (dotted vertical lines). Red and black data points are below and above the detection limit, respectively. Blue dashed lines show expected values. (**d**) qPCR standard curves for viral major capsid protein quantification. All 96-well plates are shown with letters corresponding to Experiment Name in provided data set. Source data are provided at https://github.com/benjaminwilliamknowles/Coup-de-Grace.
