# Supplementary material for: Temperate infection in a virus–host system previously known for virulent dynamics
Source: Nat Commun. 2020 Sep 15;11:4626. doi: 10.1038/s41467-020-18078-4 (PMC7493887; doi:10.1038/s41467-020-18078-4)
Supplement: Supplementary file 3 — Reporting Summary [file 41467_2020_18078_MOESM3_ESM.pdf]

nature research

Corresponding author(s): Ben Knowles, Kay Bidle: NCOMMS-20-18076-T

Last updated by author(s): Jun 15, 2020

# Reporting Summary

Nature Research wishes to improve the reproducibility of the work that we publish. This form provides structure for consistency and transparency in reporting. For further information on Nature Research policies, see our Editorial Policies and the Editorial Policy Checklist.

## Statistics

For all statistical analyses, confirm that the following items are present in the figure legend, table legend, main text, or Methods section.

| n/a | Confirmed | |
|---|---|---|
| ☐ | ☒ | The exact sample size ($n$) for each experimental group/condition, given as a discrete number and unit of measurement |
| ☐ | ☒ | A statement on whether measurements were taken from distinct samples or whether the same sample was measured repeatedly |
| ☐ | ☒ | The statistical test(s) used AND whether they are one- or two-sided<br>*Only common tests should be described solely by name; describe more complex techniques in the Methods section.* |
| ☒ | ☐ | A description of all covariates tested |
| ☒ | ☐ | A description of any assumptions or corrections, such as tests of normality and adjustment for multiple comparisons |
| ☐ | ☒ | A full description of the statistical parameters including central tendency (e.g. means) or other basic estimates (e.g. regression coefficient) AND variation (e.g. standard deviation) or associated estimates of uncertainty (e.g. confidence intervals) |
| ☒ | ☐ | For null hypothesis testing, the test statistic (e.g. $F$, $t$, $r$) with confidence intervals, effect sizes, degrees of freedom and $P$ value noted<br>*Give P values as exact values whenever suitable.* |
| ☒ | ☐ | For Bayesian analysis, information on the choice of priors and Markov chain Monte Carlo settings |
| ☒ | ☐ | For hierarchical and complex designs, identification of the appropriate level for tests and full reporting of outcomes |
| ☒ | ☐ | Estimates of effect sizes (e.g. Cohen's $d$, Pearson's $r$), indicating how they were calculated |

*Our web collection on statistics for biologists contains articles on many of the points above.*

## Software and code

Policy information about availability of computer code

| | |
|---|---|
| Data collection | Data were collected using FlowJo v7 from cytograms generated via flow cytometry using BD flow cytometry software (Accuri C6, Influx); photochemcial health assessed with FiRE in-house software; viruses counted with MxPro qPCR software; all statistics and plotting were done in R, especially with the ggplot2 package; mathematical modeling was done using Fortran; data were collected from prior publications with the online WebPlotDigitzer tool (https://automeris.io/WebPlotDigitizer/). |
| Data analysis | Data were collected using FlowJo v7 from cytograms generated via flow cytometry using BD flow cytometry software (Accuri C6, Influx); photochemcial health assessed with FiRE in-house software; viruses counted with MxPro qPCR software; all statistics and plotting were done in R, especially with the ggplot2 package; mathematical modeling was done using Fortran; data were collected from prior publications with the online WebPlotDigitzer tool (https://automeris.io/WebPlotDigitizer/). |

For manuscripts utilizing custom algorithms or software that are central to the research but not yet described in published literature, software must be made available to editors and reviewers. We strongly encourage code deposition in a community repository (e.g. GitHub). See the Nature Research guidelines for submitting code & software for further information.

## Data

Policy information about availability of data

All manuscripts must include a data availability statement. This statement should provide the following information, where applicable:

- Accession codes, unique identifiers, or web links for publicly available datasets
- A list of figures that have associated raw data
- A description of any restrictions on data availability

All empirical and theoretical data sets are being uploaded with this resubmission. We have also posted all data to a publicly accessible Github project (https://github.com/benjaminwilliamknowles/Coup-de-Grace).

# Field-specific reporting

Please select the one below that is the best fit for your research. If you are not sure, read the appropriate sections before making your selection.

☐ Life sciences ☐ Behavioural & social sciences ☒ Ecological, evolutionary & environmental sciences

For a reference copy of the document with all sections, see nature.com/documents/nr-reporting-summary-flat.pdf

# Ecological, evolutionary & environmental sciences study design

All studies must disclose on these points even when the disclosure is negative.

| | |
|---|---|
| Study description | We ran a series of cultures with or without viral infection and monitored the response in growth/death, cellular stress, and viral production. We then compared the outcome of the experiments with the predictions of theoretical models and already-published field data. |
| Research sample | Empirical experiments involved following the dynamics of Emiliania huxleyi strain CCMP374 and its virus strain EhV207 to represent environmental interactions of these organisms. Published data sets characterized the dynamics and abundances of mixed communities of E. huxelyi and EhVs in the environment. |
| Sampling strategy | Samples were randomized and blind-labeled (i.e., cultures were labeled just with random numbers). Initial experiments showed us very expected dynamics, despite being replicated (n = 3 per treatment) and sampled every day for a week (short term dynamics). In order to see if these results were robust or misleading, we then repeated the experiments ~7 times, with all treatments in more than one of these experiments, with samples replicated (generally n = 3). We were only convinced of the robustness of our results after assembling a data set of hundreds of data points, which is ten-fold or more what are commonly published in similar papers. As our experiments continued, we came to sample longer and longer dynamics, and started to undertake stratified sampling (we sampled on Fibonacci sequence days: 0, 1, 2, 3, 5, 8, 13, 21) to capture both short-term and long-term dynamics. |
| Data collection | Empirical data were collected via flow cytometry and qPCR from randomized, blind-labeled samples. The operator ran and processed the samples, but was not aware of which samples were which. |
| Timing and spatial scale | Empirical experiments were conducted under routine laboratory condition (light, temperatures) with established laboratory cultures that are continuously maintained (i.e., cultures are the same regardless of when experiments were done etc). |
| Data exclusions | Data were not excluded. |
| Reproducibility | The empirical experiments described here were repeated > 3 times (in terms of our core cell count and viral count metrics), to give us confidence in our findings, but also because we were initially surprised by our results and only came to believe them after repeated, independent experiments verified them. |
| Randomization | All samples (i.e., empirical cultures) were randomly numbered to blind data collection and processing. Cultures were also randomly placed within incubators, etc. |
| Blinding | All data collected in this study (i.e. new experimental data) was collected in a way such that the operator was unable to identify samples during acquisition (i.e., blind) -- samples were randomly numbered and only identified after data entry such that statistical analysis could be done. We don't know if published data used here were collected and analyzed in a blind manner. |

Did the study involve field work? ☐ Yes ☒ No

# Reporting for specific materials, systems and methods

We require information from authors about some types of materials, experimental systems and methods used in many studies. Here, indicate whether each material, system or method listed is relevant to your study. If you are not sure if a list item applies to your research, read the appropriate section before selecting a response.

## Materials & experimental systems

| n/a | Involved in the study | |
|---|---|---|
| ☒ | ☐ | Antibodies |
| ☐ | ☒ | Eukaryotic cell lines |
| ☒ | ☐ | Palaeontology and archaeology |
| ☒ | ☐ | Animals and other organisms |
| ☒ | ☐ | Human research participants |
| ☒ | ☐ | Clinical data |
| ☒ | ☐ | Dual use research of concern |

## Methods

| n/a | Involved in the study | |
|---|---|---|
| ☒ | ☐ | ChIP-seq |
| ☐ | ☒ | Flow cytometry |
| ☒ | ☐ | MRI-based neuroimaging |

## Eukaryotic cell lines

Policy information about cell lines

| | |
|---|---|
| Cell line source(s) | Cells of Emiliania huxelyi strain CCMP374 have been in culture for decades following isolation from the environment. |
| Authentication | NA; cells have just been kept in culture since isolation. |
| Mycoplasma contamination | NA; also unlikely given that cultures are maintained in seawater (osmotic stress). |
| Commonly misidentified lines (See ICLAC register) | NA |

## Flow Cytometry

### Plots

Confirm that:

- [x] The axis labels state the marker and fluorochrome used (e.g. CD4-FITC).
- [x] The axis scales are clearly visible. Include numbers along axes only for bottom left plot of group (a 'group' is an analysis of identical markers).
- [x] All plots are contour plots with outliers or pseudocolor plots.
- [x] A numerical value for number of cells or percentage (with statistics) is provided.

### Methodology

| | |
|---|---|
| Sample preparation | Emiliania huxelyi strain CCMP374 was grown under established laboratory conditions (light, temperature, etc). Cells were subjected to varied infection treatments by the virus strain EhV207. Cell abundances and physiological state were assessed by flow cytomtery and viral abundances by qPCR. |
| Instrument | Data were collected on BD Influx and Accuri flow cytometers. |
| Software | Data were collected with BD Influx and Accuri software. Data were extracted from the resulting flow cytograms using FlowJo v7 and parsed into spreadsheets in Excel for plotting and analyses with R. |
| Cell population abundance | No sorts were done. |
| Gating strategy | Live Emiliania huxelyi cells were identified by routinely used chlorophyll / size gates (i.e., extensively published). Live cells identified this way were then probed for staining or autofluorescence by stress-marker stains (described in full in the Methods). In post-collection processing (FlowJo v7), cells were characterized as stain-positive using routinely used gates or by gates drawn with unstained controls for reference (also described in the Methods). |

- [x] Tick this box to confirm that a figure exemplifying the gating strategy is provided in the Supplementary Information.
